# An analysis of the clinical application of paliperidone palmitate injection based on real-world

**DOI:** 10.3389/fphar.2025.1501701

**Published:** 2025-03-26

**Authors:** Ruixue Liu, Chunxiao Liu, Di Hu, Longkun Wang, Dianwei Feng, Tongxin Guo, Ying Wang

**Affiliations:** ^1^ Department of Pharmacy, Tianjin Anding Hospital, Tianjin, China; ^2^ Department of Pharmacy, Tianjin Union Medical Center, The First Affiliated Hospital of Nankai University, Tianjin, China

**Keywords:** paliperidone palmitate, schizophrenia, long-acting injectable antipsychotics, paliperidone palmitate 1-month, paliperidone palmitate 3-month

## Abstract

**Objective:**

To analyze the clinical application, adherence and relapse of paliperidone palmitate (PP) injection.

**Method:**

The information of patients treated with PP 1-month (PP1M), patients switching from PP1M to paliperidone palmitate 3-month (PP3M), and the relapse of patients after discharge from 1 January 2021 to 31 December 2023 was retrieved from the electronic medical record system. Descriptive statistics, two-tailed *t*-test and Chi-Squared test are used to process data.

**Results:**

189 patients were administered with PP1M in this study accounted for 0.88% of all inpatients. 5 (9.09%) patients were switched from risperidone long-acting injection (RLAI) to PP1M. 45 (∼23.81%) patients were switched into PP3M. The patients without relapses were twice than relapsed patients after discharge of 1 year. Patients with initial episodes administered with PP1M showed fewer relapses than patients with non-initial episodes (11.11% vs. 34.72%; ^a^ *P* < 0.05). 75 (39.68%) patients adhering to PP1M or PP3M after discharge showed fewer relapses than patients discontinuing injection (20.00% vs. 35.09%; ^b^ *P* >0.05). Besides, our results showed that the instability of disease and the non-adherence were the main factors contributing to relapse.

**Conclusion:**

The current utilization of PP1M and conversion from PP1M to PP3M is low. Patients with initial episodes with and adhering to PP injection were at lower risk of relapse. Thus, the patients’ adherence and the education about PP injections should be improved.

## Introduction

Schizophrenia is a chronic mental disorder with high relapse rate which is accompanied by psychosis and various behavioral, cognitive and social deficits (Millan et al., 2016; Kane and Correll, 2019; Donati et al., 2020). It affects approximately 21 million people with the lifetime prevalence around 0.30%–0.66% globally (Guo et al., 2021). Although clinical remission could be obtained with proper treatments, nearly 2/3 of patients who discontinue medication in one and a half years suffer from relapse which are manifested with clinical deterioration, functional impairments and treatment resistance (Kim et al., 2021; Lieberman Jeffrey et al., 2005; Takeuchi et al., 2019). Therefore, relapse mitigation or elimination is the primary endpoint for schizophrenia management.

Paliperidone palmitate (PP) is one of the long-acting injectable antipsychotics (LAIs) formulations of paliperidone, which is the major active metabolite of risperidone. The incorporation of a palitoyl ester contributes to the low water solubility, which results in the extended-release and slow diffusion into circulation after injection and the sustained plasma concentrations of paliperidone (Hough et al., 2009). PP is currently available in 3 pharmaceutical ingredients formulations: PP1M, PP3M and PP6M. PP1M formulation is smaller nanocrystal, whose half-life ranges from 25 to 49 days that could reach steady state after 9 injections (8 months). The most common adverse reactions (AR) of PP1M (incidence ≥5% and occur at least twice as often as placebo) were injection site reactions, somnolence/sedation, dizziness, akathisia, and extrapyramidal symptoms (Peters et al., 2023; Schoretsanitis et al., 2018; Titusville, 2021a). PP3M formulation applies larger nanocrystal aggregates and higher concentration to slow dissolution, whose half-life ranges from 84 to 95 days that takes >1 year to reach steady state. The most common AR of PP3M were also the injection site reaction, increased weight, headache, upper respiratory tract infection, akathisia, and parkinsonism (Peters et al., 2023; Schoretsanitis et al., 2018; Titusville, 2021b). PP6M incorporates a biodegradable polymer matrix to create microspheres releasing PP gradually over 6 months, whose half-life ranges from 148 to 159 days and the concentration of plasma paliperidone 18 months after dosing of 1,560 mg PP6M is estimated to be 18% of the average steady-state levels. The most common AR of PP6M were upper respiratory tract infection, injection-site reaction, increased weight, headache, and extrapyramidal symptoms (Peters et al., 2023; Schoretsanitis et al., 2018; Titusville, 2022).

PP3M is applied to patients who respond to PP1M for at least 4 months. PP3M demonstrates comparable safety and efficacy as PP1M, and may be applied to patients frequently due to non-frequent dosing schedule (Morris and Tarpada, 2017). Compared with PP1M, PP3M has a lower risk of relapse (Weiden et al., 2017). LAIs can improve adherence and continuity, consequently mitigating relapse, mortality whereas improving prognosis (Najarian et al., 2023; Turkoz et al., 2024; Correll et al., 2024). A study showed that LAI application lowered risk of death to around 30% compared with the oral application of the same medication and the lowest mortality was observed for paliperidone LAIs monthly (Taipale et al., 2018). An independent 10-year mirror image study confirming sustained improvements for patients completed 5 years on PP1M showed an overall reduction of 72% in the mean number and 68% in the mean length of admissions compared to the 5-year period before initiation (Pappa et al., 2023). LAIs treatment during early stage resulted in significantly improved clinical outcomes in patients with schizophrenia, especially those with initial episode and short duration (Kim et al., 2021; Abdel-Baki et al., 2020).

Thus far, despite the encouraging evidence increases, LAIs is not widely prescribed due to the reserved opinions of the patients, physicians, and payers (Parellada and Bioque, 2016). The inappropriate cost-effectiveness and cost of LAIs are limiting factors for its application in the United States and China. One study in the United States suggested that adults with schizophrenia were stabilized by extended-release of paliperidone followed by switch to paliperidone LAIs which was not cost-effective within 5 years due to higher drug costs, while PP6M was cost-effective over PP1M and PP3M (Wang et al., 2023). A cross-sectional investigation in Beijing showed that the main obstacle to widespread use of LAIs was high costs (Zhu et al., 2021). A study on the administration avenues of LAIs in schizophrenia in 15 Asian countries/regions indicated that LAIs application in China was 0.66% which was largely lower than the average (Tang et al., 2020). A study in India reported that 78.8% of patients chose oral tablets while 5% made LAIs as the first choice (Grover et al., 2019). Another study based on several European countries indicated the low application of LAIs was due to the reservations of the prescribers for seemingly more difficult-to-treat patients and those unlikely to adhere to oral medication (Llorca et al., 2018). At present, the efficacy and benefits of PP injections have been largely reported. However, there is a lack of analysis of the application of PP injections based on real-world. Our study aims to analyze the real-world application, adherence and relapse of PP injections therapy.

## Methods

This study has been reviewed and approved by the Medical Ethics Committee of Tianjin Anding Hospital. All data in the study were retrieved from the Hospital Network Information System (HIS) and electronic medical record system of Tianjin Anding Hospital from 1 January 2021 to 31 December 2023. We described the detailed information including patient characteristics: sex, age, educational background, duration of illness, hospitalization experience, smoking/alcohol use history and comorbidity (hypertension, diabetes, hyperlipidemia), dosing details (dosage, switch from PP1M to PP3M), adherence, outcomes of discharged patients (relapse/non-relapse in 1 year) and reasons of relapse. Relapse was defined as any one of the following: (1) psychiatric hospitalization because of worsening symptoms. (2) Deliberate self-injury, clinically significant suicidal. (3) Violent behavior leading to injury of another individual or property damage (Emond et al., 2019).

The inclusion criteria for patients of this study are as follows: (1) patients meet the ICD-10 standard and are diagnosed with schizophrenia. (2) Older than 18-year-old. (3) Patients have been clinically stable on receiving paliperidone extended-release or risperidone for ≥ 4-week (Simpson et al., 2015). (4) Patients were excluded if they received other antipsychotics. From 1 January 2021 to 31 December 2023, we analyzed PP1M usage in 210 patients, excluding 21 patients based on the aforementioned criteria: three patients not diagnosed with schizophrenia; one patient younger than 18-year-old; five patients were administered with paliperidone extended-release or risperidone for<4-week; five patients received only one injection during hospitalization; seven patients received other antipsychotics. Concomitant prescriptions for patients with other drugs were shown in Supplementary Table S1. Concomitant prescriptions are classified as benzodiazepines, non-benzodiazepines, antidepressants, anxiolytics and mood stabilizers.

All analyses were performed by using Microsoft EXCEL 2019. The statistical significance of the two-tailed *t*-test was set at *P* < 0.05. Qualitative variables were presented by frequency and percentage. The Chi-Squared test was used as comparing categorical variables. Variables included in the study were gender, age, education level, hospitalization experience, course of the disease, smoking/alcohol use history, and comorbidity.

## Results

### Basic characteristics of the patients

The demographic characteristics of all patients in this study were shown in Table 1. There were no statistically significant differences in age, sex, educational level, course of disease, hospitalization experience, smoking/alcohol use history and comorbidity among three groups. 189 patients in this study were composed of 101 female patients (53.44%) and 88 male patients (46.56%). Two-thirds (66.67%) of the patients were ≤44-year-old followed by those between 45-and 64-year-old (32.28%), while only two patients were ≥65-year-old (1.06%). The highest education level of the patients was graduate, which accounted for 2.65%. Patients with high school degree accounted for 32.80% which are the largest population. In term of the disease course, 5.82% of patients have suffered for over 30 years followed by those between 10 and 30 years (49.74%), those between 5 and 10 years (22.22%) and those <5 years (22.22%) sequentially. Additionally, 23.81% of patients have experienced the initial episode of hospitalization. 13.76% of patients had a history of smoking/alcohol use. 17.67% of patients had comorbidity including hypertension, diabetes and hyperlipidemia.

**TABLE 1:** Descriptive statistics of basic characteristics.

Variable	2021 (n = 74)	2022 (n = 63)	2023 (n = 52)	*χ* ^2^	*P value*
Age				5.82	0.666
≤44	49	43	34		
45–64	24	20	17		
≥65	1	0	1		
Sex				1.33	0.514
Female	39	37	25		
Male	35	26	27		
Education level				13.21	0.213
Graduate	2	2	1		
Bachelor	23	12	15		
College	11	16	9		
High school	22	23	17		
Junior	10	8	7		
Primary	6	2	3		
Course of disease				7.88	0.246
<5	20	14	8		
≥5,<10	13	17	12		
≥10,<30	34	29	31		
≥30	7	3	1		
Hospitalization experience				0.97	0.617
Initial episode	18	17	10		
Non-initial episode	56	46	42		
Smoking/Alcohol use history				3.09	0.213
Yes	8	7	11		
No	66	56	41		
Comorbidity				2.59	0.274
Yes	17	9	7		
No	57	54	45		

### PP1M prescription and its switch to PP3M

The information of PP1M prescription and its switch to PP3M was shown in Table 2. 189 of 21496 (0.88%) patients were prescribed with PP1M. Among these patients, five of 55 (9.09%) patients were switched from risperidone long-acting injection (RLAI) to PP1M. The prescription of PP1M decreased annually, which was the lowest in 2023. 45 of 189 (23.81%) patients were switched from PP1M to PP3M.

**TABLE 2:** Total PP1M prescription and the information of the switch to PP3M.

Year	PP1M (n)		Inpatients (n)	Percentage (%)	Switch from PP1M to PP3M(n)		Percentage (%)
	Male	Female			Male	Female	
2021	35	39	6,999	1.06%	11	4	20.27%
2022	26	37	6,656	0.95%	3	17	31.75%
2023	27	25	7,841	0.66%	4	6	19.23%
Total	88	101	21,496	0.88%	18	27	23.81%

### Outcome of the patients

Discharged patients administered with PP1M with/without relapse and the related relapse factors were summarized in Table 3. The number of patients without relapse is over twice that with relapse in 1 year after discharge. Treatment non-adherence is the main factor contributing to relapse (54.55%).

**TABLE 3:** The outcome and relapse factors of discharged patients from 2021 to 2023.

	2021	2022	2023	Overall
Relapse	27 (36.49%)	17 (26.98%)	11 (21.15%)	55 (29.10%)
Non-Relapse	47 (63.51%)	46 (73.02%)	41 (78.85%)	134 (70.90%)
Factors for relapse				
Disease fluctuation	8 (29.63%)	10 (58.82%)	7 (63.64%)	25 (45.45%)
Self-withdrawal	19 (70.37%)	7 (41.18%)	4 (36.36%)	30 (54.55%)

### Relapse in patients of initial episode and non-first episode after LAIs application

The relapse of discharged patients of initial episode or not were shown in Table 4. Among 189 patients recruited in this study, 45 (23.81%) patients with initial episode have been prescribed with PP1M and their relapse was significantly decreased compared with patients of non-initial episode (11.11% vs. 34.72%, ^a^ *P* < 0.05).

**TABLE 4:** Relapse in patients of initial and non-initial episodes.

Year	Initial episode		Non-initial episode	
	PP1M (n)	Relapse (%)	PP1M (n)	Relapse (%)
2021	18	4 (22.22)	56	23 (41.07)
2022	17	1 (5.88)	46	16 (34.78)
2023	10	0 (0.00)	42	11 (26.19)
Total	45	5 (11.11)	144	50 (34.72^a^)

^a^ *P* values of continuous variables for the comparison of relapse rates of patients of initial episode versus patients of non-initial episode were calculated using the *t*-test.

^a^ *P* < 0.05.

### The relapse of discharged patients with/without adherence to LAIs

The relapse of discharged patients with/without adherence to LAI was showed in Table 5. 75 of 189 (39.68%) patients continuing the LAIs therapy showed fewer relapse than patients discontinuing the therapy, though it was not significant (20.00% vs. 35.09%; ^b^ *P* > 0.05).

**TABLE 5:** The relapse rates of discharged patients with/without adherence to LAIs.

Year	Adherence to LAIs		Non-adherence to LAIs	
	Adherence (n)	Relapse (%)	Non-adherence (n)	Relapse (%)
2021	20	3 (15.00)	54	24 (44.44)
2022	31	7 (22.58)	32	10 (31.25)
2023	24	5 (20.83)	28	6 (21.43)
Total	75	15 (20.00)	114	40 (35.09^b^)

^b^ *P* values of continuous variables for the comparison of relapse rates of adherence to LAIs, versus non-adherence to LAIs, were calculated using the t-test.

^b^ *P* > 0.05.

## Discussion

Our study provides a comprehensive analysis of real-world paliperidone utilization in schizophrenia patients, encompassing its application across the entire treatment continuum. Key aspects investigated include: clinical application of PP1M and subsequent switch to the PP3M; Post-discharge adherence: persistence with injectable regimens following hospital discharge; Incidence of disease recurrence and its association with first-episode status and injection adherence. Using two-tailed t-test, we quantified the relationship between adherence to PP injections (particularly in first-episode patients) and reduced relapse risk. This real-world evidence highlights the role of LAIs optimization in improving longitudinal outcomes for schizophrenia management.

We reported the clinical application, adherence and relapse of patients treated with LAIs. In our study, more than half patients with duration >10-year were treated with LAIs which indicated its early application was needed. Several studies concluded that early initiation of LAIs showed therapeutic benefits including improved adherence, the mitigated symptoms, reduced relapse and medical cost (Kim et al., 2021; Subotnik et al., 2015; Munday et al., 2019). Another study suggested that patients with longer duration, particularly >10 years, were conferred with higher risk of relapses despite the satisfied adherence. Although evidence for the effectiveness of LAI antipsychotics in the early stage of schizophrenia is increasing, they are mostly administered at the late stage after the occurrence of non-adherence due to psychotic relapses. Therefore, LAIs should be considered at the early stages to improve the prognosis of patients.

The prescription of PP1M was low and decreased annually according to our study. However, in clinical and real-world studies, though PP1M and PP3M were more effective than antipsychotics in oral application in delaying relapse, symptomatic control and functional improvements, their clinical use was limited (Turkoz et al., 2024). Only 5% of patients used LAIs in China which was lower than in India (15.8%) (Guo et al., 2021; Tang et al., 2020). In European countries, the prescription of LAIs is between 20% and 40% (Tang et al., 2020; Barnes et al., 2009; Llorca et al., 2013). The obstacles to LAIs application include insufficient understanding of LAIs usage by the physicians, the lack of patient insight into the disease and the treatment, and the limited healthcare policy (Guo et al., 2021). Therefore, it is crucial to formulate the clinical guidelines of LAI antipsychotics and provide well education of LAIs for physicians and patients.

Among the patients using PP1M, 5 (9.09%) patients were converted from RLAI. A prospective multi-centre open-label intervention study lasting 6 months showed that one-third of risperidone long-acting therapy (RLAT)-treated patients achieved >50% improvement in PANSS score after switching to PP1M (Schreiner et al., 2015). One retrospective longitudinal cohort study found that compared with RLAI, patients treated with PP1M had lower hospitalization, emergency department visits and hospitalization costs (Joshi et al., 2016). Moreover, one-quarter of the patients switched from PP1M to PP3M in our study due to the increasing evidence of the benefits and efficacy of PP3M. Patients switching from PP1M to PP3M showed fewer relapse, improved adherence and long-acting effect (Weiden et al., 2017; Emond et al., 2019; Li et al., 2002; Joshi et al., 2017; DerSarkissian et al., 2018; Lin et al., 2021a). Therefore, it is recommended that patients at stable stage should turn to longer interval injection therapy to improve compliance and prognosis whereas reduce relapse and care burden. Notably, the decision to switch to longer interval injection therapy should primarily depend upon clinical manifestations to achieve optimal efficacy and safety.

LAI effectively reduced the relapse especially in patients with initial episode. Relapse is common in patients with schizophrenia (80% of patients relapse within 5 years) (Ma et al., 2023). It was reported that the application of LAI risperidone after the initial episode of schizophrenia showed pronounced advantages over oral risperidone according to clinical outcomes (Subotnik et al., 2015; Nuechterlein et al., 2022). Guidelines suggested that LAI antipsychotics could be applied to patients with initial episode or patients at the early stage of schizophrenia (Galletly et al., 2016; Norman et al., 2017; Barnes et al., 2019). Thus, early initiation of antipsychotic treatment is crucial which could improve the prognosis of schizophrenia.

It was reported that relapse occurred in patients with well adherence to treatment (Alphs et al., 2016). This is consistent with our results (Table 5). However, non-adherence to treatment is the key contributor to relapses in schizophrenia. Factors associated with non-adherence to antipsychotic treatments include illness factors (e.g., poor insight into illness, cognitive impairment), medication factors (side effects, regimen complexity, perceived lack of efficacy), physician/service factors (communication, therapeutic alliance), patient factors (stigma, past history of adherence, attitudes to medication and illness) and caregiver factors (attitudes to medication and illness, ability to supervise/remind patient about medication) (Haddad et al., 2014). A comprehensive review of recent literature in 2002 showed that the mean nonadherence rate is 49.5% (Lacro et al., 2002). The discontinuation of oral anti-psychotics in chronic schizophrenia has been estimated to be 74% after 18-month of therapy (Lieberman Jeffrey et al., 2005). A meta-analysis showed that patients initiated on an LAI were 89% more likely to be adherent to medication compared with those on an oral anti-psychotics (Lin et al., 2021b). A real-world retrospective investigation demonstrated that the LAIs cohort exhibited significantly higher medication adherence compared to the oral anti-psychotics cohort, with a greater proportion of days covered (69% vs. 64%), higher medication possession ratio while on therapy (82% vs. 64%), and improved persistence rates (85% vs. 80%) (Dickson et al., 2023). Hence, the rate of non-adherence has changed in the last 30 years due to LAIs. The ways to advance adherence include: (1) policies should be adopted to popularize the publicity and education of LAIs. (2) Psychiatrists’ knowledge of LAIs and health education for patients should be provided. (3) Physicians should have proper perception about LAIs and positive attitudes toward patients and LAIs therapy. (4) It was necessary to strengthen communication between physicians and patients. Physicians should make sure that patients understand what LAIs is and why LAIs formulations are offered.

The limitations of this study should be noted: (1) due to the limited research time (the tracing time of patients’ relapse was short) and clinical data, our study was a limited study and the strength of causal linkage should be improved with more cases in the future. (2) Relapse was defined by re-hospitalization while the information of some patients transferred from other hospitals could not be retrieved.

## Conclusion

Our study analyzes the schizophrenia patients with clinical application of PP injection in the real-world. We found the application of PP injection is relatively rare and LAI anti-psychotics should not be limited to patients with low non-adherence. Physicians and patients should show positive attitude toward LAIs, initiate LAIs at the early stage and switch to longer interval injection therapy at the proper time. Our study indicates that the LAIs therapy deserves further research and evaluation.

## Data Availability

The original contributions presented in the study are included in the article/Supplementary Material, further inquiries can be directed to the corresponding author.
