# Supplementary material for: An analysis of the clinical application of paliperidone palmitate injection based on real-world
Source: Front Pharmacol. 2025 Mar 26;16:1501701. doi: 10.3389/fphar.2025.1501701 (PMC11978662; doi:10.3389/fphar.2025.1501701)
Supplement: Supplementary file 1 [file Table1.docx]

Supplementary Table 1. Drugs concomitant prescribed for patients.

| Concomitant medications | Drug classes |
| --- | --- |
| Benzodiazepines | Intermediate-acting drug (alprazolam, estazolam, oxazepam, lorazepam, nitrazepam)  Long-acting drug (clonazepam) |
| non-Benzodiazepines | Zolpidem, Zopiclone, Eszopiclone, Zaleplon |
| Antidepressant | SSRIs (fluoxetine, fluvoxamine, sertraline, Paroxetine)  SNRIs ( duloxetine)  SARIs (trazodone)  NassAs (mirtazapine) |
| Anxiolytic | Tandospirone |
| Mood stabilizer | Sodium valproate, lithium carbonate |
| Antihypertensive drugs | Nifedipine, Valsartan,Enalapril |
| Antidiabetic drugs | Metformin, Acarbose |
| Hypolipidemic drugs | Simvastatin |
